# Tree shrews (*Tupaia belangeri*) exhibit novelty preference in the novel location memory task with 24-h retention periods

**DOI:** 10.3389/fpsyg.2014.00303

**Published:** 2014-04-14

**Authors:** Jayakrishnan Nair, Marlene Topka, Abbas Khani, Manuela Isenschmid, Gregor Rainer

**Affiliations:** ^1^Visual Cognition Laboratory, Department of Medicine, University of FribourgFribourg, Switzerland; ^2^Fribourg Center for Cognition, University of FribourgFribourg, Switzerland

**Keywords:** spatial novelty, spatial memory, habituation, depression, animal models, 24 h retention period, major depressive disorder, depressive disorder

## Abstract

Novelty preference is pervasive in mammalian species, and describes an inherent tendency to preferentially explore novelty. The novel location memory task studied here assesses the ability of animals to form accurate memories of a spatial configuration, consisting of several identical objects placed within an arena. Tree shrews were first familiarized with a particular object configuration during several sessions, and then an object was displaced during a test session. Tree shrews exhibited enhanced exploration when confronted with this novel configuration. The most reliable indicator associated with novelty preference was an enhancement in directed exploration towards the novel object, although we also observed a non-specific overall increase in exploration in one experiment. During the test session, we also observed an exploration of the location, which had previously been occupied by the displaced object, an effect termed empty quadrant. Our behavioral findings suggest multiple stages of spatial memory formation in tree shrews that are associated with various forms of behavioral responses to novelty. Reduced novelty preference has been linked to major depressive disorder in human patients. Given the established social conflict depression model in tree shrews, we anticipate that the study of the neural circuits of novelty preference and their malfunction during depression may have implications for understanding or treating depression in humans.

## INTRODUCTION

Novelty seeking has been identified as one of six major human personality dimensions, and refers to the tendency of individuals to exhibit a preference for novelty (Cloninger, 1986). Reduced novelty seeking has been linked to depression; for example it is correlated to the severity of symptoms in patients suffering from major depressive disorder (Celikel et al., 2009). Reduced novelty seeking can be considered a core symptom of depression, and it is closely related to rigid evaluative patterns and reduced coping flexibility that also characterize the depressive state (Kato, 2012; Jacobs, 2013). In the Cloninger personality scheme, novelty seeking is composed of the four aspects exploratory excitability, impulsiveness, extravagance, and disorderliness. Of these aspects, only exploratory excitability, describing the inclination to be excited by and preferentially explore novelty, has been shown to be significantly reduced in patients suffering from major depression (Hansenne et al., 1999). This is fortuitous, because exploratory preference for novelty can be studied in animals. Indeed, in rodents, novelty preference has already been indirectly linked to depression-like symptoms in a recent study (Stedenfeld et al., 2011). In this study, wild-type Sprague–Dawley rats were bred for exhibiting high locomotor responses to novel environments. When subsequently tested on their tolerance to chronic mild stress, these animals were more resilient than controls. Since depression often appears as a result of stressors, this observation provides a link between novelty preference and depression at the behavioral level.

The literature on animal models of depression is extensive, and investigators have employed various behavioral paradigms including chronic stress, learned helplessness, and social defeat to produce behavioral states, mostly in rodent laboratory animals, that bear similarities to depression in humans (Deussing, 2006; Krishnan and Nestler, 2011). A particularly interesting approach has been the psychosocial stress paradigm in the tree shrew (von Holst, 1969; Fuchs and Flügge, 2002; Fuchs and Flïugge, 2006; Wang et al., 2011), which is based largely on the continued olfactory and visual contact of a subordinate male with a dominant male after a brief period of social conflict. Under these conditions, the subordinate develops symptoms including reduction in hippocampal volume, endocrine changes, and abnormal core body temperature regulation that closely mirror depressive symptoms in humans. Furthermore, the tree shrew model of depression is sensitive to a number of anti-depressant drugs used in humans to treat major depressive disorder (van der Hart et al., 2005; Schmelting et al., 2013). Available evidence thus suggests that psychosocial stress induced depression in tree shrews may be a depression model with a high internal and external validity (Fuchs, 2005), thus making it particularly interesting to uncover the associated changes of neural circuits that occur in this close relative of primates (Cao et al., 2003) during depression.

The established link between novelty preference and depression, together with the well-documented tree shrew animal model of depression, suggest that exploring how tree shrews respond to novelty may produce insights with relevance for understanding particular aspects of major depressive disorder in human patients. We have previously demonstrated that tree shrews exhibit novelty preference in the novel object recognition task (Khani and Rainer, 2012). This task, which assesses exploration of a novel object introduced into a familiar arena, is a commonly used behavioral paradigm for testing behavioral novelty responses (Ennaceur and Delacour, 1988; Antunes and Biala, 2012) Less frequently used is a protocol where the spatial configuration of elements in an arena is modified without changing their composition, a task referred to as novel location recognition test (Takahashi et al., 2013). An interesting aspect of this task is that, unlike the novel object recognition task, it appears to involve hippocampal circuits of memory formation (Barker and Warburton, 2011) that are known to be important for spatial navigation and memory (O’Keefe and Nadel, 1978). Both tasks are based on the observation that exploration time of an identical environment decreases or habituates over time, and the introduction of a novel object or a novel spatial configuration results in a rebound of exploratory activity, whose magnitude depends on various factors including for example stimulus complexity, species, and retention period (Brennan et al., 1984; Poucet et al., 1988).

In the current study, we were interested in exploring the sensitivity of tree shrews to a change in spatial configuration of an environment, and characterizing aspects of tree shrew behavior during the performance of a novel location memory task.

## MATERIALS AND METHODS

### ANIMALS

Adult female tree shrews (*Tupaia belangeri*) aged between 2 and 4 years were housed in 6 m^3^ cages that were connected through a tube to a nesting box, with generally up to five animals per cage. They were kept in a temperature-controlled room and maintained on a standard 12-h light/dark cycle (light on at 08:00). Food and water were available *ad libitum*. All experimental procedures were in compliance with European and applicable Swiss regulations.

### APPARATUS

**FIGURE 1:**
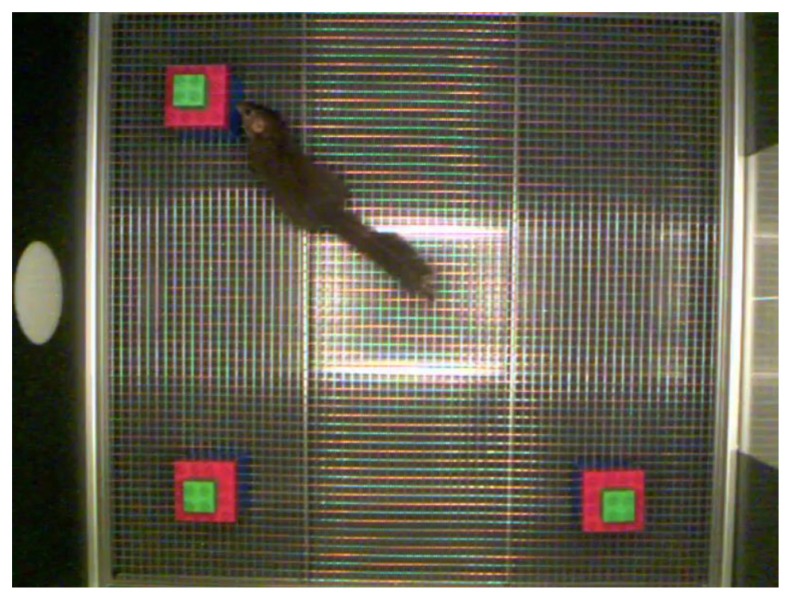
**Top view of the experimental setup, illustrating the relative size of the objects and the tree shrew during exploration of one object**.

Our testing apparatus was composed of a rectangular arena (60 cm × 60 cm) covered by a pyramidal ceiling with a camera mounted on the top at a height of about 70 cm above the arena floor. Arena walls were made of black plastic panels and the arena was illuminated with LED lights strips fixed on the ceiling. The arena contained numerous spatial cues including the arena entrance and a sliding door of light gray color opposite to the entrance. The arena was placed in a quiet testing room close to the animal room. The objects to be remembered were objects made of white plastic container with a red lid (approx. 7 cm × 7 cm × 10 cm) in Experiment 1 and “Lego” objects (approximately 7 cm × 10 cm × 10 cm) composed of about 15 stacked “Duplo” blocks of mixed colors in Experiments 2 and 3. All objects were affixed to the arena floor using metal screws positioned below the objects and inaccessible to the animals. A top view of the experimental setup, illustrating the relative size of the object and the tree shrew during exploration of one of the objects is shown in **Figure 1**. Arena and objects were cleaned with 70% ethanol between behavioral sessions to avoid any olfactory trails (Wakita et al., 2008; Ostrander et al., 2011). In all experiments the object displacement location was counterbalanced between left and right to avoid confounds due to potential spatial bias. All the experiments were performed between 8.30 am and 12.30 pm, corresponding to the morning peak activity of the tree shrews.

## PROCEDURE: EXPERIMENT 1

**FIGURE 2:**
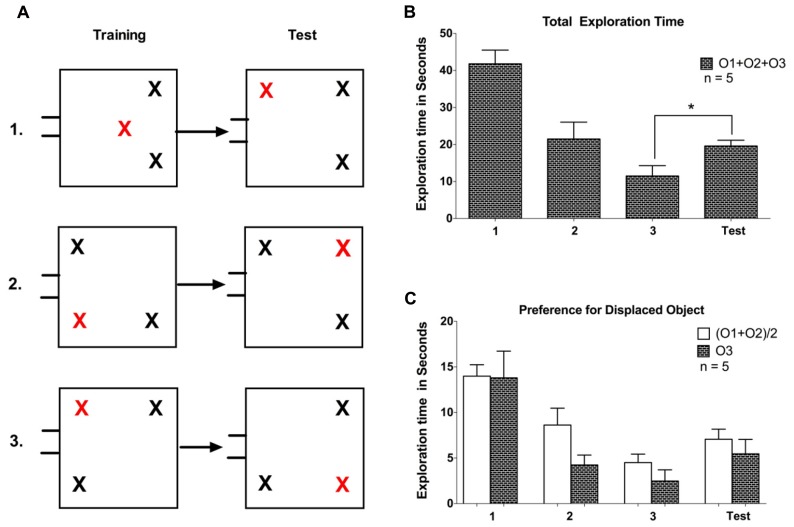
**(A)** Representation of training and test phases for the three configurations used in Experiment 1. All the objects were identical; the red cross marks the to-be displaced object in the trainings sessions and the displaced object during the test session. **(B)** The total exploration time spent on exploring all objects (O1 + O2 + O3) on the three training session and subsequent test session. O3 is the displaced object. **(C)** Comparison of exploration for the displaced object O3 with the average of the non-displaced objects (O1 + O2)/2. Error bars denote the SEM (*P < 0.05).

The experiment consisted of three phases: arena familiarization; training (Object familiarization); and a test session. In all phases, one of the five task-naïve tree shrews used in this experiment, was carried to the test room inside the nesting box, which was connected to the arena using a flexible tube. The gate was opened and the animal was given the opportunity to enter and explore the arena, while being able to freely commute between the arena and the nesting box through the connecting tube. The arena familiarization sessions were considered complete when 10 min elapsed after the first time the animal had entered the arena. After 3 days of familiarization to the empty arena, three identical objects (white plastic container with red lid) were placed in the arena in the training phase lasting three sessions. Again, animals could commute freely between the arena and the nesting box and now explore objects inside the arena. Every animal was allowed 7-10 min (starting when the animal first entered the arena) to explore the objects. We used three different initial configurations in this experiment, including two where one object was displaced to the opposite corner and another configuration where a central object was displaced to one corner (see **Figure 2A**). No apparent differences in behavior were observed between these configurations, so data are treated together. On the test day, when one of the objects was displaced to a new location, tree shrews were again brought to the arena and were allowed to explore the objects for about 7-10 min. After each session, the gate was closed while the animal was inside the nesting box, and the animal was returned to its home cage and released.

## PROCEDURE: EXPERIMENT 2

The second experiment was conducted about 4 weeks after Experiment 1, using the previous five animals and two additional naïve ones. The same three phases were performed (arena familiarization, object training, and test session). However, in contrast to the first experiment, we here conducted two test sessions.

**FIGURE 3:**
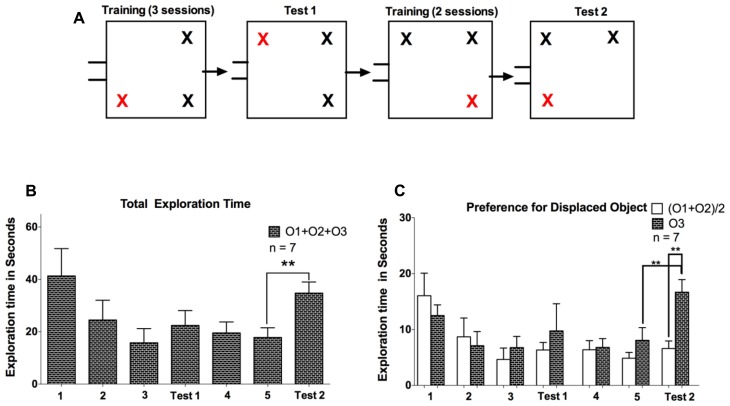
**(A)** Representation of training and subsequent test phases in Experiment 2. Two training phases composed of three and two sessions were conducted; each followed by a test session in which one object was displaced. O3 marks the displaced object. **(B)** The total exploration time spent exploring all objects (O1 + O2 + O3) during the training and test sessions in Experiment 2. **(C)** Comparison of exploratory activity for the displaced object O3 to the average of the non-displaced objects (O1 + O2)/2. Error bars denote the SEM (**P < 0.01).

After 2 days of arena familiarization (10 min each), we performed 3 days of object training (5 min each) with three identical objects made of Lego in the same object configuration for all seven animals. After a 24-h retention period, we then conducted the first test session with the displacement of one of the objects (**Figure 3A**). This object configuration was maintained for another training phase lasting for 2 days, followed by the second test session after another 24-h retention period, again with the displacement of one of the objects. In contrast to Experiment 1, animals were not able to return to their nesting box during the tasks, to ensure that each animal spent an equal amount of time in the arena. This measure was taken because we observed in Experiment 1 that once animals left the arena, they were hesitant to return.

## PROCEDURE: EXPERIMENT 3

We conducted this experiment to examine whether increasing the number of consecutive days with object training sessions would result in improved novelty preference on the test day. Five tree shrews, which had previously been used in Experiments 1 and 2, and four naïve tree shrews were tested. After 4 days of arena familiarization for 10 min each, the animals were trained for five consecutive days for a duration of 5 min on the first two days and 10 min on the last 3 days. The test phase was performed on the sixth day with the displacement of one object to a new location (see **Figure 5A**). Also, animals were not able to return to their nesting box during the tasks.

## DATA ANALYSIS AND STATISTICS

All phases of all experiments were conducted in a closed system and the experimenter did not handle animals or otherwise intervene during the experiment. The experimenter was monitoring animal behavior online on a computer screen through the connected camera. Video was analyzed offline for scoring using the iMovie software (version 9.0.9 Apple Inc.) or VLC player, to quantify exploratory behavior separately for each object during the first five min that the animal spent inside the arena. Pointing of the nose at an object at a distance of less than 1 cm or touching an object were considered criteria for object exploration. We determined both the time spent exploring each object and the total object exploration time. Video scoring was performed independently by two observers, which yielded similar results. All data are expressed as mean ± SEM. Statistical analyses used were student’s paired *t*-tests, repeated measures analysis of variance (ANOVA), and linear regression. A probability level less than 0.05 was considered as statistically significant.

## RESULTS

### EXPERIMENT 1

To evaluate spatial memory retention in tree shrews, we first allowed animals to explore a spatial configuration of three objects during three training sessions conducted on subsequent days (see **Figure 2B**). We observed a gradual decline in exploratory activity, demonstrated by a significant reduction in the time animals spent exploring the objects (*F* = 18.84, *P* < 0.01, one-way repeated measures ANOVA). On the subsequent test day, we introduced a change in the spatial configuration, such that one object (O3) was displaced to a previously empty location. Animals exhibited a rebound of exploratory activity, demonstrated by a significant increase in exploration time between the test day and the previous (third) training day (*P* < 0.05, *t* = 4.4, *df* = 4, paired samples *t*-test). We next asked whether the increased exploratory activity on the test day was directed mostly at the displaced object (O3; see **Figure 2C**). We observed no exploration advantage of the displaced object over the average value for the other two (O1, O2) unchanged objects (*P* > 0.1, *t* = 0.64 *df* = 4, paired samples *t*-test). Taken together, following three training sessions we observed a general increase in exploratory activity on the test day for naïve animals, and no exploration preference for the displaced object.

### EXPERIMENT 2

Here our aim was to extend the results of Experiment 1 and evaluate the effects of additional training sessions on exploratory motivation. After a rest period of about 4 weeks following Experiment 1, we subjected the same group of five animals plus two naïve animals to five training sessions, as well as two test sessions after the third and fifth training day. The spatial configuration of objects of the first test session was maintained for training sessions four and five, such that animals experienced two changes in spatial configuration during this experiment, occurring after the third and fifth training session (see **Figure 3A**). Animals exhibited a statistical trend consistent with a gradual decline of exploratory activity during the first three training days (*F* = 3.31, *P* = 0.07, one-way repeated measures ANOVA), and a comparison in total exploration time between the first and third training sessions reached statistical significance (*P* < 0.05, *t* = 2.81, *df* = 6, paired samples *t*-test). In contrast to Experiment 1, we did not observe a significant rebound in exploratory activity on the first test session, conducted after the third training day (*P* = 0.45, *t* = 0.81, *df* = 6, paired samples *t*-test), although six of seven animals did in fact exhibit an increase in exploration time (see **Figure 3B**). One possible reason for this difference between Experiments 1 and 2 is a potentially reduced effectiveness of the training sessions when animals are subjected to repeated experiments. Based on a power analysis we conclude that insufficient statistical power thus cannot be ruled out as a potential source of the non-significant rebound in exploration time after three training sessions in Experiment 2.

**FIGURE 4:**
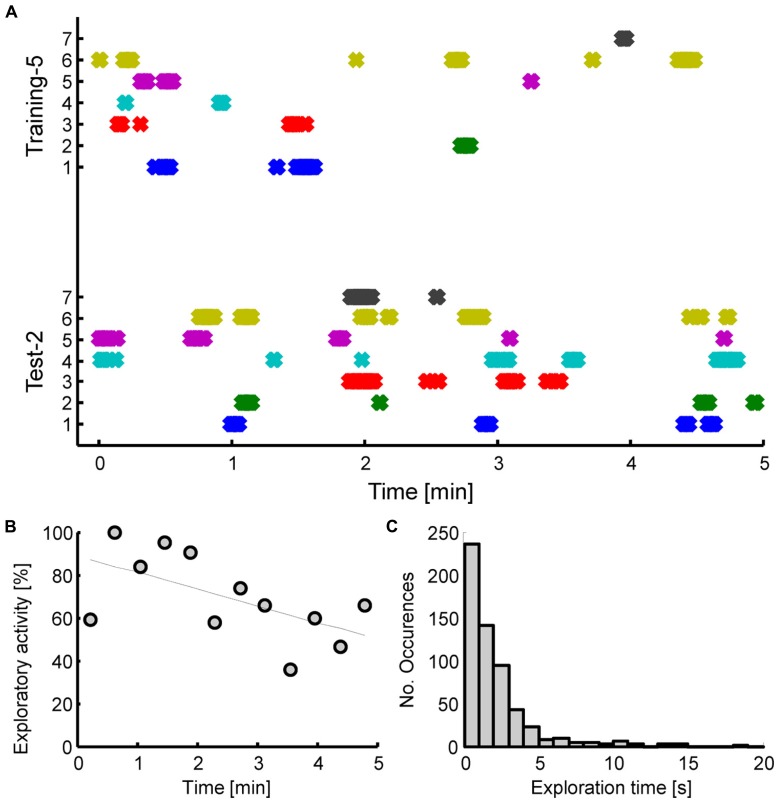
**(A)** Exploration times of individual tree shrews for the displaced object during the second test session (bottom panel), and as a control the corresponding times for the same object prior to displacement during the last training session (top panel). Animals 1-5 were experienced and had already participated in previous similar experiments whereas animals six, seven were task naïve. A cross symbol is plotted for each 100 ms interval that the animal spent exploring. Continuous shaded regions thus represent multiple overlapping symbols and correspond to continued exploration. **(B)** Time course of overall exploration time for all objects during the first 5 min of exploration for all training and test sessions considered together. **(C)** Distribution of duration of exploration for individual contacts with the target objects (O1, O2, or O3).

After an additional two training sessions, and thus a total of five training sessions in Experiment 2, animals did exhibit a robust rebound of exploratory activity during the test session (*P* < 0.01, *t* = 5.25, *df* = 6, paired samples *t*-test). The increased exploration time was largely directed towards the displaced object (O3). To demonstrate this, we performed a two-way ANOVA with object [levels: (O1 + O2)/2, O3] and interval (levels: training 5, test 2) as factors, which yielded significant main effects (*P* < 0.01) and interaction (*P* < 0.05). *Post hoc* tests, using Holm-Sidak correction for multiple comparisons indicated significance of (O1+O2)/2 vs. O3 exploration time on the test day (*P* < 0.01), as well as O3 exploration time on training 5 vs. test day (*P* < 0.01).The displaced object was thus explored longer than the average of the two unchanged objects (O1, O2) on the test day, and also longer than the corresponding object prior to displacement on the last training day (see **Figure 3C**). Analysis of the number of contacts with the objects produced similar results to the temporal analysis, with tree shrews showing higher number of object contacts for the displaced object. For the comparison between the displaced object on the test day to the same object prior to displacement on the last training day, we examined in detail the exploratory behavior of each individual animal during the 5 min exploration interval (see **Figure 4A**). This analysis illustrates that exploration occurred at the beginning, middle, and end of the interval, and that exploratory patterns were variable across animals. For example, animal 3 tended to explore relatively infrequently, whereas animal 6 spent much more time exploring. Novelty preference, defined as the average time difference spent exploring the displaced compared to the control object, was estimated for each min of the interval yielding average values of -2.1, 21.4, 25.0, 17.9, and 23.9 s for the first to the last interval min, respectively. This indicates that novelty preference did not appear to be concentrated at the beginning of the interval but tended to be distributed throughout the exploration period. An analysis of all exploratory periods across the entire experiment including all training and test sessions (a total of 49 sessions from seven animals) revealed that while exploration was on average greatest at the beginning of the interval, it did continue during the entire interval, reaching a value of about 60% at the end. Exploratory activity was significantly negatively correlated with the time during the interval (*R*^2 ^= 0.35, *P* < 0.05), as shown in **Figure 4B**. Finally, the duration of individual explorations was distributed as shown in **Figure 4C**, with most explorations lasting less than 5 s. The mean duration of each exploration was 2.12 ± 0.10 s, and corresponding median value was 1.40 s. Taken together, five training sessions led to a robust enhancement in general exploratory activity that was largely directed at the novel object, in animals that had previous experience with spatial memory experiments.

### EXPERIMENT 3

**FIGURE 5:**
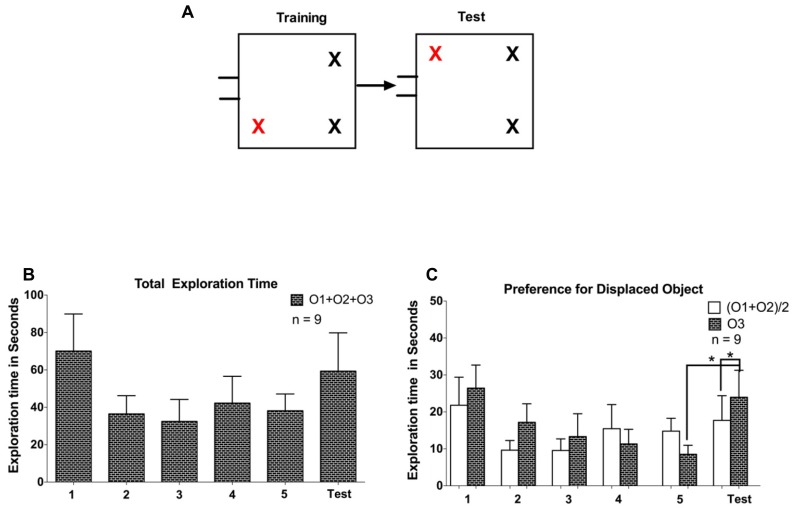
**(A)** Representation of experiment phases in Experiment 3. **(B)** Total exploration time spent exploring all objects (O1 + O2 + O3). O3 represents the displaced object. **(C)** Comparison of exploratory activity for the displaced object O3 to the average of the non-displaced objects (O1 + O2)/2. Error bars denote the SEM (*P < 0.05).

**FIGURE 6:**
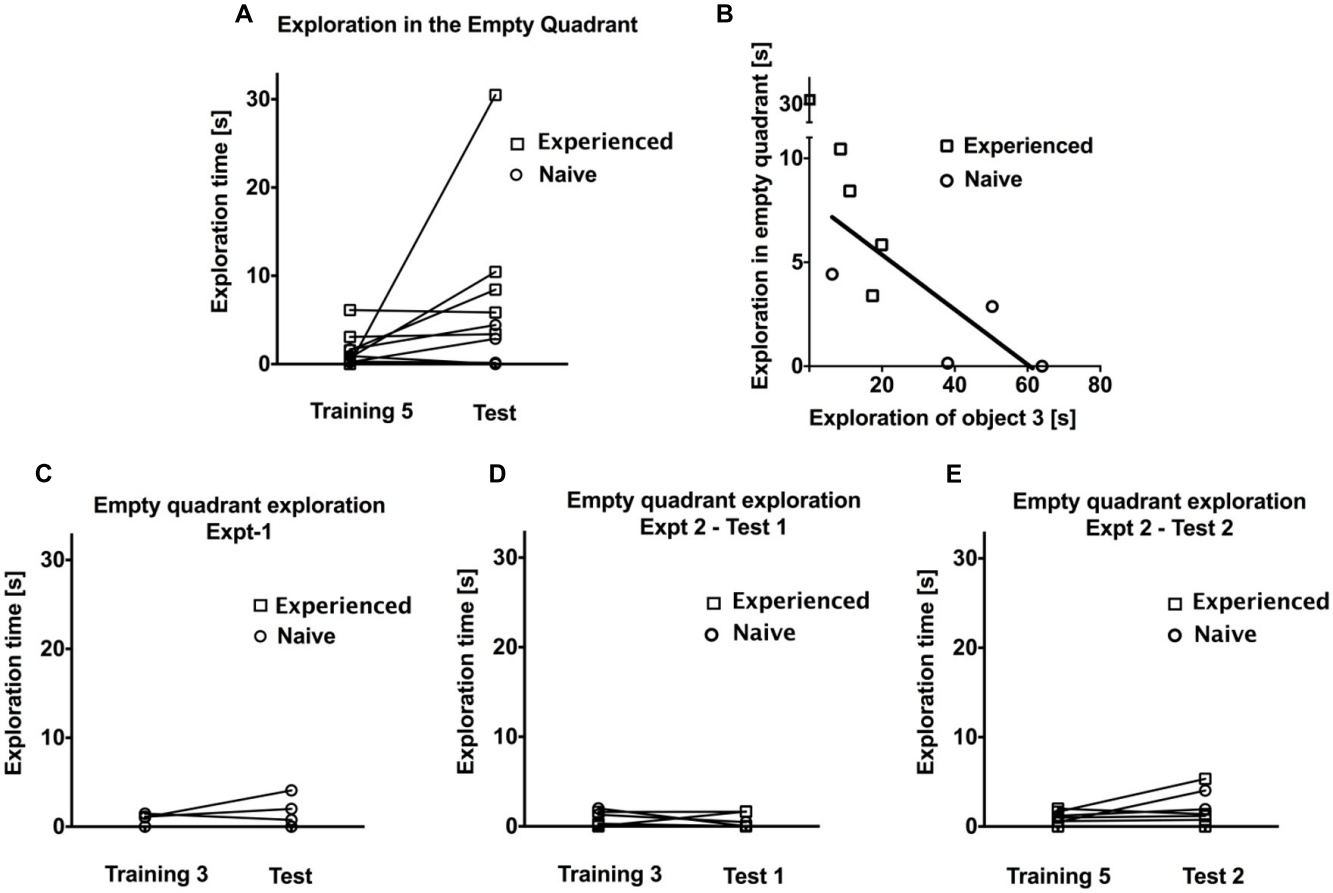
**(A)** Comparison of exploratory activity of the empty quadrant during the test session and the corresponding area on the prior training session in Experiment 3 for experienced and naive animals. Round symbol shows the naive animals and square symbol shows the experienced ones. **(B)** Linear regression demonstrating that animals, which spent considerable time exploring the empty quadrant, tended to devote little exploration to the displaced object on the test day in Experiment 3. **C–E** shows the comparison of exploratory activity of the empty quadrant during the test session and the corresponding area on the prior training session in Experiment 1, Experiment 2 for test 1 and prior training session, and test 2 and prior training session, respectively, for experienced and naive animals.

Finally, we wanted to examine the effect of five continuous training sessions without an intervening test session on exploration time to a change in spatial configuration. Four naïve animals, as well as five animals that also completed Experiments 1 and 2, participated in Experiment 3 (see **Figure 5**). As in Experiments 1 and 2, there was a gradual decrease in exploratory activity over the course of the first three training sessions (*F* = 6.02, *P* < 0.05, one-way repeated measures ANOVA). There was no significant difference in exploration time between training sessions three and five (*F* = 0.38, *P* > 0.1, one-way repeated measures ANOVA), suggesting that exploration time has reached an asymptotic value from about the third training session onwards. Surprisingly, we did not observe a general rebound in the time spent on object exploration on the test day (*P* > 0.1, *t* = 1.41, *df* = 8, paired samples *t*-test; see **Figure 5B**). However, as in Experiment 2, animals did on average spend more time exploring the displaced object, both compared to the average of the unchanged objects during the test session (*P* < 0.05, *t* = 2.926, *df* = 8) as well as compared to the corresponding object during the previous training session (*P* < 0.05, *t* = 2.785, *df* = 8; see **Figure 5C**). We observed a peculiar behavior in some animals that might partly explain the lack of a general rebound in object exploration activity during the test session: an exploration of the empty quadrant that had previously been occupied by the displaced object was evident in several animals, particularly those that had participated already in Experiments 1 and 2. Naïve animals by contrast did not tend to exhibit this behavior. This is illustrated in **Figure 6A**, which plots exploration times of the empty quadrant during the test session to the corresponding exploration time on the fifth training session, showing that in particular three tree shrews with previous task experience exhibited considerable empty quadrant exploration. This suggests that there was in fact additional exploration occurring during the test session that is not included in the object exploration time, contributing to the non-significant rebound in total exploration on the test day. Interestingly, animals that spent a lot of time exploring the empty quadrant tended to show little exploration of the displaced object (see **Figure 6B**). This is demonstrated by an anti-correlation between these exploration times (*P* = 0.064, R^2 ^= 0.41, linear regression on all data; *P* < 0.05, *R*^2 ^= 0.58, linear regression without the outlier animal that spent over 30 s exploring the empty quadrant during the test session). Given the appearance of empty quadrant exploration in Experiment 3, we went back to examine whether this behavioral strategy also occurred during Experiments 1 and 2. We found that empty quadrant exploration was very rare in these prior experiments, as is illustrated in **Figures 6C–E**. Empty quadrant exploration time remained below 6 s in all of these experiments; in contrast to Experiment 3 where three of the experienced animals exhibited empty quadrant exploration times above 6 s (compare **Figure 6A**). For all three experiments, we repeated the exploration rebound analysis between the last training and test days, including the empty quadrant exploration time (O1 + O2 + O3 + EQ), and found identical results to those reported above for (O1 + O2+ O3), namely significant rebound in exploration time in Experiment 1 and Experiment 2: training day 5 vs. test 2. The comparison of (O1 + O2 + O3 + EQ) for Experiment 3 does not reach statistical significance, although a trend towards significance is apparent (*P* = 0.07, *t* = 2.05, *df* = 8) that was not seen for the O1 + O2 + O3 parameter (*P* > 0.1, *t* = 1.41, *df* = 8).

Taken together, across experiments we found that preference for the displaced object appears to be a more reliable indicator for spatial configuration memory than total exploration time, probably as a result of empty quadrant exploration, particularly for task-experienced animals.

## DISCUSSION

We have shown that tree shrews are sensitive to novelty in a novel location recognition task after multiple training sessions and with a 24-h interval between training and test session. Multiple training sessions are necessary for robust novelty preference: Across the three experiments, we observed that three object training sessions, as employed in Experiments 1 and 2, do not lead to robust location memory formation. In Experiment 2, we observed neither a general nor a directed increase in exploratory activity to the novel spatial configuration on the test day, whereas in Experiment 1 we saw only a general increase that likely results from a fragile memory of the object training configuration. In tree shrews, five object training sessions appear necessary for robust location memory and novelty preference, as evidenced by the significant exploratory activity directed towards the displaced object in the novel location in Experiments 2 and 3. Interestingly, a general increase in exploration was seen in Experiment 2, whereas it did not reach statistical significance for Experiment 3. We suggest that this is probably due to the empty quadrant exploration exhibited by some individual tree shrews during the test session in Experiment 3. These animals spent time exploring the space where the displaced object had been during the previous training session, reflecting an accurate location memory, but this time is not included in the total exploration time. Our findings suggest that empty quadrant exploration, which has been previously documented in spatial memory tasks (Thinusblanc et al., 1987; Xavier et al., 1991), is mostly exhibited by individuals that have already participated in previous spatial novelty experiments. Empty quadrant exploration may thus reflect an advanced stage of memory formation and task knowledge, and particular attention should be paid to this effect when evaluating exploration time in spatial novelty paradigms.

Novelty preference indicates that a memory of the training configuration has been maintained for a retention interval of 24 h in the tree shrew, suggesting an involvement of long-term memory. In the rat, location novelty preference tasks, including the task employed in our study (Ennaceur et al., 1997; Dix and Aggleton, 1999) as well as related paradigms such as the object-in-place task (Barker and Warburton, 2009), are typically conducted with short retention intervals ranging from 5 min upto 1 h. Indeed, there is evidence suggesting that rats do not exhibit significant habituation in exploratory activity during repeated exposures to an unchanged environment, when these exposures are separated by 8-10 h (Poucet et al., 1988). This lack of habituation suggests that spatial memory has not been maintained and the animals are treating the environment as novel after a 10 h period. Interestingly, the same study showed that both hamsters and to a lesser degree gerbils do exhibit significant habituation in terms of the number of object contacts under the same conditions. It has been suggested that these differences may reflect species-specific strategies in gathering and processing of information in the environment (Poucet et al., 1988). One possibility is that the enhanced time of exploration per contact observed in hamsters (1.87 s) compared to rats (1.21 s) might lead to superior memory encoding and thus at least partly underlie the different habituation patterns. Our results are generally consistent with this hypothesis, since tree shrews exhibit both long-term spatial memory maintenance and relatively high exploration time per contact (2.12 s) as compared to the other species above. Tree shrews thus appear capable to encode spatial memories and maintain them for 24-h periods, probably at least in part to the robust memory formation that is related to relatively long exploration time devoted to the objects on each contact during the object training sessions.

A related aspect of tree shrew explorative behavior concerns the dynamics of exploration within each session. We have shown that tree shrews continue to explore the objects in the arena during a 5 min interval, and exploration time falls off approximately linearly reaching about 60% of the initial maximum at the end of the interval. Novelty preference exhibits a similar time course during the 5 min exploration interval and our findings suggest that it certainly does not appear to be limited to the beginning of the exploratory period. This observation is in significant contrast to related findings in the rat, where exploratory behavior shows a peak during the first min and novelty-related effects are limited also mostly to this first min of exploration time, sometimes extending to the second min but not beyond (Dix and Aggleton, 1999). Thus, tree shrews and rats exhibit marked differences in overall exploration and novelty preference, with rats showing massed effects restricted to the first minute or two of the exploration interval, and tree shrews utilizing the entire 5 min interval, and possibly beyond, for continued exploration.

The development of spatial memory formation is a subject of current interest in developmental psychology, where in particular allocentric, rather than egocentric, spatial memory abilities have been linked to the development of hippocampal circuits (Konkel and Cohen, 2009; Ribordy et al., 2013), consistent with animal studies demonstrating a dependence of allocentric spatial memory on an intact hippocampus (Morris et al., 1982) as well as with the allocentric reference frame that the hippocampus is thought to employ for spatial navigation (O’Keefe, 1990, 1991). Allocentric spatial memory is generally studied by providing multiple entrances into an arena or disorienting the participant prior to arena access, so that egocentric cues cannot be used to remember particular configurations or object placements inside the arena. Since our arena only had a single entrance and animals always entered by the same path, we cannot exclude that egocentric cues might contribute to the preferential exploration during the test session where one of the objects was displaced to a novel location. Evidence for an egocentric strategy would be, if novelty preference in tree shrews was based mostly on an initial approach and prolonged exploration of the displaced object immediately after the animal enters the arena. However, our data show that tree shrews did not use this strategy, instead continuing to explore the displaced object during the entire 5 min interval while moving around the arena. Tree shrews also did not appear to immediately target the displaced object for exploration (see for example **Figure 6A**); also generally favoring an allocentric memory interpretation. This interpretation is consistent with the finding that the novel location recognition task is disrupted by hippocampal damage (Barker and Warburton, 2011), and supports the notion that tree shrews rely on hippocampal circuits during memory formation and resultant novelty preference in the location memory task studied here.

The apparent dependence of the novel location memory task on an intact hippocampus suggests that it may test a form of memory that corresponds to episodic memory in humans, a part of the declarative memory system. On the other hand, other evidence suggests that novelty preference in fact depends more strongly on implicit rather than explicit, declarative memories (Snyder et al., 2008). In either case, novelty preference can be considered as a form of priming, in the sense that the priming stimulus, i.e., the prior object training session(s), exerts an influence on behavior during the test session. Priming is generally considered to depend on short-term memory, which of course cannot bridge the 24-h delay between training and test sessions. It has been proposed that nevertheless, priming underlies habituation and novelty responses (Terry, 1979), in that the contextual information such as the transfer and connection of the nesting box to the arena serve as cues to recall the memorized stimulus, i.e., the configuration of the training arena, to short-term memory, where it can have an impact on subsequent explorative behavior.

Taken together, our findings validate a behavioral paradigm in the tree shrew, thereby extending the known behavioral repertoire of this species and complementing previous work in visual psychophysics and decision making (Petry et al., 1984; Callahan and Petry, 2000), effects of stress on memory tasks (Ohl and Fuchs, 1998, 1999; Ohl et al., 1998) and object novelty preference (Khani and Rainer, 2012). Our findings indicate that tree shrews are sensitive to novelty in the novel location memory task, and thus studying the neurophysiological activity of associated circuits of memory formation, including the hippocampus, may be useful to reveal the neural circuits underlying novelty preference. Such knowledge can provide a basis for studying potential malfunctions of this circuit during depression in the tree shrew, which may provide useful insights relevant for understanding or treating major depressive disorder in human patients.

## Conflict of Interest Statement

The authors declare that the research was conducted in the absence of any commercial or financial relationships that could be construed as a potential conflict of interest.
